# Streptococcal Mannose Phosphotransferase System Component IID Is a Novel RANK‐Binding Osteoclastogenic Factor

**DOI:** 10.1002/advs.77282

**Published:** 2026-08-19

**Authors:** Chaeyeon Park, Yeongkag Kwon, Yeonjin Lim, Jeong Woo Park, Hyun Jung Ji, Dongwook Lee, Jueun Lee, Sung‐Ho Yun, Sungho Jeong, Ho Seong Seo, Kun Cho, Cheol‐Heui Yun, Ok‐Jin Park, Seung Hyun Han

**Affiliations:** ^1^ Department of Oral Microbiology and Immunology, and Dental Research Institute School of Dentistry Seoul National University Seoul Republic of Korea; ^2^ Research Division For Radiation Science Korea Atomic Energy Research Institute Jeongeup Republic of Korea; ^3^ Center For Research Equipment Korea Basic Science Institute Ochang Republic of Korea; ^4^ Department of Agricultural Biotechnology, and Research Institute of Agriculture and Life Sciences Seoul National University Seoul Republic of Korea; ^5^ Department of Microbiology and Immunology College of Dentistry and Research Institute of Oral Sciences Kangwon National University Gangneung Republic of Korea

**Keywords:** inflammatory bone diseases, mannose phosphotransferase system component IID, osteoclast differentiation, RANK, streptococci

## Abstract

Streptococci, Gram‐positive opportunistic pathogens, can cause inflammatory bone diseases, yet their virulence factors remain unclear. We investigated streptococcal components contributing to bone pathology. Unlike *Staphylococcus aureus*, streptococci and lipoprotein‐deficient strains induced comparable bone destruction and osteoclastogenesis in mice via a TLR2‐independent pathway. A comparative Q Exactive Plus mass spectrometric analysis of streptococcal and staphylococcal membrane proteins led us to identify mannose phosphotransferase system component IID (Man‐PTSIID) as a novel osteoclastogenic factor. Recombinant Man‐PTSIID and synthetic peptides containing conserved amino acid sequences of Man‐PTSIIDs found in various streptococci bound to the receptor activator of nuclear factor‐κB (RANK), as demonstrated by an SPR analysis. Structural modeling and molecular docking analysis, together with full‐length Man‐PTSIID binding assays, demonstrated an interaction between Man‐PTSIID and RANK. Streptococcal Man ‐ PTSIID promotes terminal osteoclast maturation and multinucleation following RANKL priming via the RANK signaling pathway, rather than mimicking native RANKL or functioning as an independent RANK agonist. Consistently, the osteoclastogenic effects induced by streptococci were not observed in Man‐PTSIID‐deficient mutant strains. Collectively, these findings suggest that Man‐PTSIID is a novel, differentiation stage‐dependent osteoclastogenic factor contributing to terminal osteoclast differentiation and plays a critical role in bone pathology during streptococcal infections.

## Graphical abstract

Streptococcal mannose phosphotransferase system component IID (Man‐PTSIID) is identified as a novel RANK‐binding osteoclastogenic factor. By directly binding to RANK and activating NF‐κB  independently of TLR2, Man‐PTSIID drives osteoclastogenesis and inflammatory bone destruction, uncovering an unexpected microbial mechanism underlying streptococcal bone pathology.

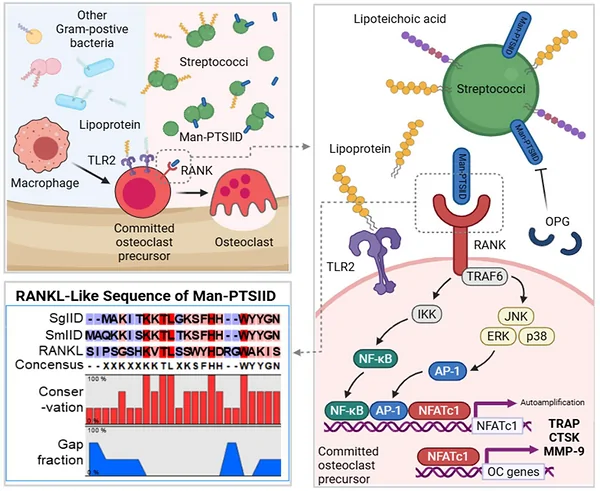

## 1 Introduction

Bone is a dynamic organ that undergoes constant remodeling to maintain its density and strength [1]. The bone remodeling process is orchestrated by the function of osteoclasts and osteoblasts. When the differentiation of osteoclasts overwhelms that of osteoblasts, bone diseases such as osteoporosis, osteomyelitis, and periodontitis occur [2]. Osteoclasts are differentiated from hematopoietic stem cells by two key osteoclastogenic factors, macrophage‐colony stimulating factor (M‐CSF) and the receptor activator of nuclear factor‐κB (RANK) ligand (RANKL) [3]. Once RANKL engages with RANK, downstream signals, including mitogen‐activated protein kinase (MAPK), NF‐κB, and nuclear factor of activated T cells 1 (NFATc1) are activated, producing mature osteoclast differentiation [4, 5]. RANKL–RANK engagement can be negatively regulated by the decoy receptor osteoprotegerin (OPG), which is mainly produced by osteoblasts [6].

Streptococci are a genus of Gram‐positive bacteria that primarily colonize the oral cavity as members of the normal commensal microbiota. However, under certain conditions, some species can act as opportunistic pathogens, disseminate through the bloodstream, and cause various diseases [7, 8, 9]. *Streptococcus gordonii* is well‐known to induce bone resorption by enhancing osteoclast differentiation and reducing osteoblast differentiation [10]. Cariogenic streptococcal infection induced dental caries and alveolar bone loss in rats [11]. Moreover, clinical studies have shown that streptococci cause bone and joint infections [12, 13, 14]. Streptococci are frequently found in the lesions of patients with lumbar osteomyelitis, septic arthritis, and apical periodontitis [12, 13, 14]. Despite the available research, the molecular mechanisms of their pathogenesis and specific treatments against streptococcus‐induced bone diseases remain poorly defined. Therefore, research to uncover the pathogenesis of streptococcal bone diseases is of critical importance for mitigating disease manifestations.

Upon bacterial infection, the recognition of pathogen‐associated molecular patterns by pattern recognition receptors is crucial for triggering inflammation and bone destruction [2]. For instance, lipoproteins, especially those from Gram‐positive bacteria, are recognized by Toll‐like receptor (TLR) 2 in the host cell and a major bone destructive factor upregulating osteoclast differentiation [15]. A lipoprotein‐deficient mutant of *Staphylococcus aureus* induced bone resorption much less than wild‐type *S. aureus* [15]. Moreover, extracellular vesicles derived from *Filifactor alocis* preferentially activated TLR2 and induced osteoclastogenesis [16]. On the other hand, little is known about the major virulence factors of streptococci and how they affect the regulation of bone metabolism.

The phosphotransferase system (PTS) is prevalent in bacteria but not in humans, and a specific family of PTS, mannose‐PTS (Man‐PTS), is notably abundant in Gram‐positive bacteria found on mucosal surfaces [17]. Man‐PTS components IIC and IID are transmembrane proteins responsible for transporting mannose into bacteria [17]. Interestingly, both IIC and IID components of the Man‐PTS also act as receptors for bacteriocin and a penetrating channel for bacteriophage lambda DNA [17]. Moreover, Fan et al. demonstrated that gluconate‐PTS from *Enterococcus faecalis* accelerates inflammatory cytokine production and colitis in murine models [18]. Although it is well‐established that streptococci can induce bone resorption, the streptococcal component that primarily contributes to bone destruction remains unknown. Therefore, in this study, we examined whether Man‐PTS is associated with the inflammatory bone diseases caused by streptococci.

## 2 Materials and Methods

### 2.1 Reagents and Chemicals

*Streptococcus mutans* ATCC 25175 and *Bacillus subtilis* ATCC 6633 were purchased from American Type Culture Collection (Manassas, VA, USA). *Streptococcus oralis* KCTC 13048, *Ligilactobacillus ruminis* KCTC 5781, *Lactiplantibacillus plantarum* KCTC 10887BP, and *Streptococcus sanguinis* KCTC 3284 were obtained from the Korean Collection for Type Cultures (Jeongeup, Korea). *Streptococcus sobrinus* KCOM 3633 and *Streptococcus mitis* KCOM 1295 were obtained from the Korean Collection for Oral Microbiology (Gwangju, Korea). *Staphylococcus epidermidis* NCCP 14768 was obtained from the National Culture Collection for Pathogens (Cheongju, Korea). *S. gordonii* CH1 and *S. aureus* RN4220 were kindly provided by Prof. Paul Sullam (University of California, San Francisco, CA, USA) and Prof. Bok‐Luel Lee (Busan National University, Busan, Korea), respectively. *S. aureus* USA300 was obtained from the Nebraska Transposon Mutant Library (Omaha, NE, USA). Lipoprotein‐deficient mutant (Δ*lgt*) strains of *S. gordonii* CH1 and *S. aureus* RN4220 were prepared as previously described [15, 19]. Using homologous recombination, Δ*lgt* strain of *S. mutans* ATCC 25175 was generated as previously described [19]. Recombinant murine M‐CSF was obtained from JW CreaGene (Seongnam, Republic of Korea). RANKL, OPG, and RANK were purchased from R&D Systems (Minneapolis, MN, USA). The synthetic peptides SgIID‐N20_5‐24_ (TKKTLGKSFHHWYYGNLTCF), SmIID‐N20_7‐26_ (SKKTLTKSFHHWYYGNLTCF), SmIID‐N11_8‐18_ (KKTLTKSFHHW), SmIID‐N7_8‐14_ (KKTLTKS), and SmIID‐N5_10‐14_ (TLTKS) were manufactured via chemical solid‐phase synthesis by AbClon (Seoul, Republic of Korea). Alpha‐minimum essential medium (α‐MEM) and fetal bovine serum were purchased from Gibco (Paisley, UK). Dulbecco's modified Eagle's medium (DMEM), penicillin/streptomycin, and trypsin‐EDTA were purchased from Hyclone (Logan, UT, USA). A tartrate‐resistant acid phosphatase (TRAP) staining kit for osteoclasts was obtained from Cosmobio (Tokyo, Japan). The endotoxin removal kit was obtained from Sigma‐Aldrich (St. Louis, MO, USA). Antibodies specific to NFATc1 were obtained from BioLegend (San Diego, CA, USA). Antibodies specific to extracellular signal‐regulated kinase (ERK), p38, c‐Jun N‐terminal kinase (JNK), phospho‐ERK, phospho‐p38, and phospho‐JNK were obtained from Cell Signaling Technology (Beverly, MA, USA). Antibodies specific to β‐actin were obtained from Santa Cruz Biotechnology (Santa Cruz, CA, USA). Horseradish peroxidase (HRP)–conjugated anti‐rabbit IgG and anti‐mouse IgG were obtained from Southern Biotech (Birmingham, AL, USA). Tryptic soy broth (TSB), Todd Hewitt broth (THB), de Man, Rogosa and Sharpe (MRS) medium, and yeast extract were purchased from BD Biosciences (San Diego, CA, USA). Luria Bertani (LB) medium was obtained from LPS Solution (Daejeon, Republic of Korea).

### 2.2 Preparation of Heat‐Inactivated Bacteria

The use of bacteria was approved by the Institutional Biosafety Committee (IBC) of Seoul National University (Approval No. SNUIBC‐R200526‐2). *S. gordonii*, *S. mutans*, *S. oralis*, *S. sanguinis*, *S. sobrinus*, and *S. mitis* were cultured in THB supplemented with 0.5% yeast extract at 37°C and grown to the mid‐log phase. *B. subtilis* were grown in TSB at 37°C to the mid‐log phase. *L. plantarum* and *L. ruminis* were grown in MRS at 37°C to the mid‐log phase. *S. aureus* RN4220, *S. aureus* USA300, and *S. epidermidis* were cultured in LB at 37°C and grown to the mid‐log phase. The bacteria were washed with phosphate‐buffered saline (PBS) and inactivated at 70°C for 2 h. Complete inactivation was confirmed by plating on proper agar plates and culturing at 37°C for more than 24 h. No bacterial colony was observed.

### 2.3 Extraction of Membrane Proteins

Membrane proteins were isolated from wild‐type (WT) and lipoprotein lipidation/maturation‐defective strain (Δ*lgt*) strains of *S. gordonii*, *S. mutans*, and *S. aureus* using the Triton X‐114 phase separation method, as previously described [15, 20], which is widely used for the enrichment of bacterial membrane‐associated proteins prior to proteomic analysis. Briefly, bacterial pellets were suspended in TBS and disrupted by sonication. The bacterial lysates were suspended in TBS buffer containing 2% Triton X‐114 and incubated at 4°C for 2 h. The bacterial lysates containing Triton X‐114 were phase‐separated by centrifugation and further incubated with an equal volume of TBS at 37°C for 15 min. After three iterations of the previous step, the Triton X‐114 phase was collected, mixed with methanol, and incubated at −20°C overnight. The precipitated membrane proteins were dissolved in 10 mM octyl‐β‐glucopyranoside in PBS.

### 2.4 Mass Spectrometric Analysis

Each Triton X‐114 extract (3 µg) was resolved by SDS‐polyacrylamide gel electrophoresis (PAGE) using a 12% gel. The gel was stained with Coomassie blue at room temperature for 1 h. After staining, the entire gel lane was excised and subjected to in‐gel digestion with enzymes such as trypsin and chymotrypsin. The resulting peptides were ionized by ion spray and scanned with a mass spectrometer (Orbitrap Exploris 240, Thermo Fisher Scientific, MA, USA) to obtain survey full‐scan mass spectra (from 40 to 6 000 *m/z*). The maximum mass resolution was *R* = 240 000 with a 1 Hz scan, and the mass accuracy was less than 5 ppm root mean square error with external calibration.

### 2.5 In Silico Docking Analysis of RANK and Man‐PTSIID

The engagement between mouse RANK and streptococcal Man‐PTSIID was modeled with the HADDOCK 2.4 web server. The mouse RANK structure was the AlphaFold model of UniProt O35305 obtained in mmCIF format. The *S. gordonii* Man‐PTSIID structure was the AlphaFold model of UniProt A8AZE6, and the *S. mutans* Man‐PTSIID structure was predicted with AlphaFold3 from the coding sequence of GenBank AOCB01000009.1. Two docking schemes were applied. In the first scheme, full‐length RANK was paired with Man‐PTSIID. RANK residues that contact RANKL within 5 Å in the mouse RANKL‐RANK complex (PDB 3ME2) were set as active residues, and the conserved N‐terminal region of Man‐PTSIID was set as active residues on the partner. In the second scheme, the RANK ectodomain and the N‐terminal region of Man‐PTSIID were extracted and docked under the same restraint definition so that membrane‐proximal regions were excluded from the search. HADDOCK grouped the water‐refined models into clusters and ranked the clusters by HADDOCK score. For each run the cluster with the best HADDOCK score was selected for analysis, and this cluster held the largest number of models, with 117 models for the full‐length *S. gordonii* complex, 28 for the full‐length *S. mutans* complex, and 8 for the ectodomain complex. Each docked complex was superposed onto chain R of 3ME2 with the matchmaker tool of ChimeraX 1.12, which placed the RANKL footprint of 3ME2 onto the docked receptor. RANK residues within 5 Å of the docked Man‐PTSIID and RANK residues within 5 Å of the RANKL chain of 3ME2 were collected, and their overlap defined the shared interface. HADDOCK score, Z‐score, buried surface area, and the component energies were recorded for every complex.

### 2.6 Generation of the Man‐PTSIID‐Deletion (Δpts) Mutant Strains

Using homologous recombination, Δ*pts* strains of *S. gordonii* and *S. mutans* were generated under IBC and living modified organism guidelines. In brief, the upstream and downstream flanking regions of the target gene were amplified by polymerase chain reaction (PCR). The PCR products were digested with the appropriate restriction enzyme (*Kpn*I, *Xho*I, *BamH*I, and *Not*I) and then ligated into the corresponding restriction enzyme sites of pC326 plasmids. Each plasmid was transformed into *S. gordonii* CH1 or *S. mutans* ATCC 25175 by the natural transformation method [21].

### 2.7 Overexpression of Man‐PTSIID Protein and 6×His Tag Pulldown Assay

For the overexpression of Man‐PTSIID proteins derived from *S. gordonii* (SgPTSIID) and *S. mutans* (SmPTSIID), each sequence was amplified with primers containing the *BamH*I and *Ava*I restriction enzyme sites. Those PCR amplicons were then inserted into pET‐21a plasmids to conjugate the 6×His tag. The plasmids were transformed into *Escherichia coli* DH5α or *E. coli* BL21(DE3), and the bacteria were selected using LB agar plates containing ampicillin. *E. coli* BL21(DE3) containing the plasmid expression vectors were cultured and treated with 0.2 mM isopropyl β‐D‐1‐thiogalactopyranoside for another 16 h and then harvested. The bacterial pellets were suspended in lysis buffer and lysed by sonication. The supernatants were incubated with Ni‐NTA agarose beads at 4°C for 2 h. The 6×His tagged Man‐PTSIID proteins were eluted using elution buffer containing up to 250 mM imidazole. Endotoxin levels in the purified recombinant SgPTSIID and SmPTSIID proteins were measured using a *Limulus Amebocyte* Lysate assay (Pierce Chromogenic Endotoxin Quant Kit, Waltham, MA, USA) and were determined to be 0.0049 EU/µg and 0.0051 EU/µg, respectively. In a separate experiment, HEK293T cells overexpressing full‐length RANK were lysed and incubated with 6×His tagged full‐length Man‐PTSIID proteins. The protein mixture was immunoprecipitated using an anti‐RANK antibody, followed by immunoblot analysis using an anti‐His antibody to detect bound Man‐PTSIID.

### 2.8 Surface Plasmon Resonance (SPR) Analysis

The SPR experiments were performed using an iMSPR‐Pro/A instrument (iCLUEBIO, Seongnam, Republic of Korea). Soluble recombinant RANK protein (50 µg/ml) was immobilized on a COOH‐Au sensor chip in 5 mM sodium acetate buffer (pH 4.0). A reference flow without the RANK protein was used for background subtraction. Each peptide analyte was diluted in HEPES buffered saline with Tween 20 (HBST) buffer (2‐fold dilution from 1 to 0.125 µM) and then injected to flow over the reference or active chip surfaces at a flow rate of 10 µl/min, and the response unit was measured. The binding kinetics between RANK and each analyte were analyzed with iMSPR‐Pro/A Evaluation software (iCLUEBIO) using a 1:1 binding model.

### 2.9 In Vivo Bone Resorption Assay

Animal experiments were approved by the Institutional Animal Care and Use Committee of Seoul National University (Approval No. SNU‐211018‐5). Six‐week‐old male C57BL/6 mice were purchased from DooYeol Biotech (Seoul, Republic of Korea). The mice were intraperitoneally injected twice with 8 mg of heat‐inactivated bacteria at a 4‐day interval, following a previously established murine calvarial bone loss model [15]. After 7 days, the right femur of each mouse was dissected and fixed in 4% paraformaldehyde. Then, the femurs were scanned with micro‐computed tomography (micro‐CT; Skyscan 1272, Bruker, Kontich, Belgium). Femurs were scanned at 70 kV, 142 µA, 1 mm AI filter, and 7 µm per image pixel size. The reconstructed three‐dimensional images were analyzed using CTAn and CTVol software (Bruker/Skyscan). Regions of interest were defined within the trabecular bone compartment, and standard bone morphometric parameters, including bone volume (BV/TV), trabecular number (Tb.N), trabecular thickness (Tb.Th), and trabecular separation (Tb.Sp), were quantified. Three‐dimensional visualization of trabecular bone structures was generated using CTVol software. In another experiment using a mouse calvarial implantation model, a collagen sheet soaked with 4.5 mg of heat‐inactivated bacteria was implanted on the mouse calvaria, as previously described [15]. After 7 days, the calvarial bone was obtained and scanned with the micro‐CT (60 kV, 166 µA, 1 mm AI filter, and 7 µm per image pixel size). Three‐dimensional images of calvarial bone were obtained by using the Skyscan program. The resorbed area was analyzed using ImageJ software (National Institutes of Health, Bethesda, MD, USA).

### 2.10 Osteoclast Differentiation

Bone marrow cells were isolated from the femurs and tibiae of a mouse and incubated in α‐MEM supplemented with 5 ng/ml M‐CSF for 24 h. Non‐adherent cells were obtained and further differentiated for 4 days into bone marrow‐derived macrophages (BMMs) with 30 ng/ml M‐CSF. The BMMs were plated onto a 96‐well cell culture plate at 2.5 × 10^4^ cells/300 µl/well and incubated with 30 ng/ml M‐CSF and 20 ng/ml RANKL for 2 days to differentiate them into committed osteoclasts. The cells were then differentiated by 30 ng/ml M‐CSF for 24 h in the presence of heat‐inactivated bacteria, Triton X‐114 extracts, peptides, or recombinant proteins. The mature osteoclasts were fixed and stained with a TRAP staining kit. TRAP‐stained cells with three or more nuclei were enumerated in a microscopic analysis.

### 2.11 Enzyme‐Linked Immunosorbent Assay (ELISA)

Cell culture supernatants of mature osteoclasts were collected. The protein levels of IL‐6 and TNF‐α in the supernatants were measured using ELISA kits (BioLegend) following the manufacturer's instructions.

### 2.12 Western Blotting

Committed osteoclasts treated with heat‐inactivated bacteria or Man‐PTSIID were lysed with HEPES lysis buffer, and the protein contents of the supernatants were quantified using a bicinchoninic acid assay. Equal amounts of proteins were then subjected to SDS‐PAGE, as described previously [22]. The gel‐separated proteins were transferred onto polyvinylidene difluoride membranes (Millipore, Bedford, MA, USA) that were blocked with 5% skim milk in TBST for 1 h and then incubated with antibodies specific to NFATc1, ERK, phospho‐ERK, JNK, phospho‐JNK, p38, phospho‐p38, or β‐actin at 4°C overnight. Each membrane was further incubated with HRP–conjugated secondary antibodies specific to mouse or rabbit IgG for 1 h. The immunoreactive bands were detected using a GeneGnome XRQ system (Syngene, Cambridge, UK).

### 2.13 Transient Transfection and Reporter Gene Assay

HEK‐293 cells expressing FLAG‐tagged RANK proteins were kindly provided by Prof. Soo Young Lee (Ewha Womans University, Seoul, Republic of Korea). The cells were selected using DMEM containing 125 µg/ml Zeocin and 3 µg/ml Blasticidin. The cells (1 × 10^6^ cells/2 mL/well) were plated onto a 6‐well culture plate in DMEM overnight and transfected with 1.2 µg of pNF‐κB‐luciferase plasmids (Promega, Madison, WI, USA) using Attractene reagent (QIAGEN, Germantown, MD, USA) for 6 h in serum‐starved conditions. The medium was replaced with fresh DMEM, and the cells were incubated for another 24 h. The transfected cells (1 × 10^5^ cells/200 µl/well) were plated onto a white 96‐well plate and stimulated with heat‐inactivated bacteria for 24 h. They were lysed, and luciferase activity was analyzed using a Bright‐Glo luciferase assay system (Promega, Madison, WI, USA).

### 2.14 Statistical Analysis

All data are shown as mean values ± standard deviations from triplicate experiments unless otherwise stated. Treatment groups were compared with an appropriate control group, and the statistics were analyzed using Student's *t*‐test. Statistical significance was set at *p* < 0.05.

## 3 Results

### 3.1 WT and ∆lgt Mutant Streptococcal Strains Induce Bone Destruction to Similar Extents

Bacterial lipoproteins are generally known to be potent osteoclastogenic factors, as lipoprotein lipidation/maturation‐defective strain (*Δlgt*) of *S. aureus* induces significantly less bone loss than its WT [15]. To investigate whether the lipoproteins of streptococci are also essential for the induction of bone destruction, we intraperitoneally injected heat‐inactivated WT and Δ*lgt* strains of *S. gordonii*, *S. mutans*, or *S. aureus* into mice. Unlike those treated with *S. aureus*, the mice treated with the WT and Δ*lgt* strains of *S. gordonii* or *S. mutans* exhibited similar bone mass (Figure 1A). Trabecular bone volume (BV/TV), trabecular thickness (Tb.Th.), and trabecular number (Tb.N.) were decreased in the groups treated with WT *S. gordonii*, *S. mutans*, or *S. aureus*, compared with the PBS‐injected group. Interestingly, the Δ*lgt* strains of *S. gordonii* and *S. mutans* also decreased BV/TV, Tb.Th., and Tb.N. about the same as their WTs. In contrast, the Δ*lgt* strain of *S. aureus* did not reduce those parameters (Figure 1B–D). Trabecular separation (Tb.Sp.) did not change significantly in any group (Figure 1E). We also implanted collagen sheets soaked with heat‐inactivated bacteria on mouse calvaria and analyzed calvarial bone. WT and Δ*lgt* strains of *S. gordonii* and *S. mutans* showed no difference in bone resorption area, whereas *S. aureus* showed a substantial change in its WT and Δ*lgt* strains (Figure 1F,G). These results suggest that the lipoproteins of streptococci are not the major factor responsible for bone destruction, unlike *S. aureus*, even though inflammatory responses may at least partially affect the extent of bone destruction.

**FIGURE 1:**
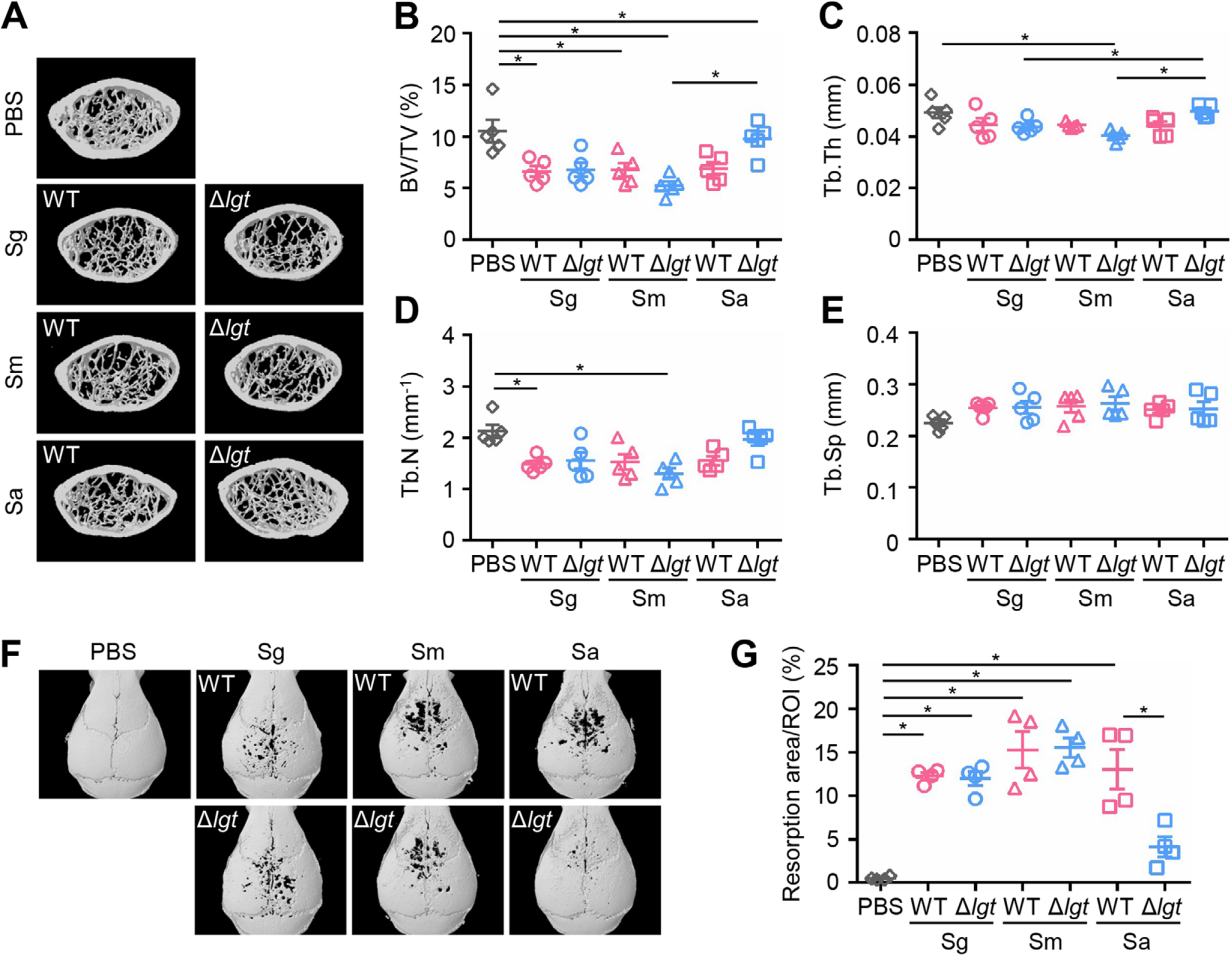
WT and ∆lgt streptococcal strains induce similar amounts of bone destruction. (A–E) C57BL/6 mice (*n* = 5 per group) were intraperitoneally injected with 8 mg of heat‐inactivated WT or Δ*lgt S. gordonii*, *S. mutans*, or *S. aureus* twice, with a 4‐day interval between injections. On day 8, the mice were sacrificed, and (A) femur images and (B–E) trabecular bone parameters were obtained using SkyScan CT analyzer. (F, G) Collagen sheets soaked with 4.5 mg of heat‐inactivated bacteria were implanted on mouse calvaria (*n* = 4 per group). (F) After 7 days, the calvarial bone was scanned by micro‐CT. (G) The resorption area of the mouse calvaria was analyzed using ImageJ. Sg, heat‐inactivated *S. gordonii*; Sm, heat‐inactivated *S. mutans*; Sa, heat‐inactivated *S. aureus*; BV/TV, trabecular bone volume; Tb.Th., trabecular thickness; Tb.N., trabecular number; Tb.Sp., trabecular separation; ROI, region of interest. One‐way ANOVA followed by Dunnett's multiple‐comparison test was performed for pairwise comparisons among groups. *p < 0.05.

### 3.2 Streptococci Augment Osteoclast Differentiation Through a TLR2‐independent Signaling Pathway

To examine the effects of those bacteria on osteoclastogenesis, we treated committed osteoclasts with *S. gordonii*, *S. mutans*, or *S. aureus*. In accordance with the in vivo data, the Δ*lgt* strains of *S. gordonii* and *S. mutans* increased the number of TRAP‐positive multinucleated cells (MNCs) equivalent to the WT level in a dose‐dependent manner, unlike *S. aureus* (Figure 2A, B). To elucidate the signaling pathway of osteoclastogenesis, a representative osteoclastogenic cascade, MAPK, and representative transcription factor, NFATc1 [23], were measured in the cell lysate. The *Δlgt* strains of *S. gordonii* and *S. mutans* retained the ability to induce phosphorylation of ERK, p38, and JNK, showing overall MAPK activation patterns comparable to those of their WT strains (Figure 2C). In contrast, the *S. aureus* *Δlgt* strain exhibited reduced ERK phosphorylation, whereas p38 and JNK phosphorylation remained similar to those of the WT strain. NFATc1 expression was not lower in these treatments with the Δ*lgt* strains of *S. gordonii* and *S. mutans*, which differed from *S. aureus* (Figure 2D). Since bacterial lipoproteins are recognized by TLR2 [15], we verified the presence of  lipoproteins in both WT and TLR2^−/−^ cells. *S. gordonii* and *S. mutans* augmented osteoclastogenesis similarly in both WT and TLR2^−/−^ cells (Figure 2E). However, IL‐6 levels were lower in TLR2^−/−^ macrophages than in WT cells in all bacteria‐treated groups (Figure 2F), implying that the TLR2 signaling pathway is irrelevant to the specific streptococcal factor that induces osteoclast differentiation. Those findings allowed us to posit that streptococcus‐induced osteoclastogenesis is mediated through interactions between committed osteoclasts and streptococcal molecules other than lipoproteins.

**FIGURE 2:**
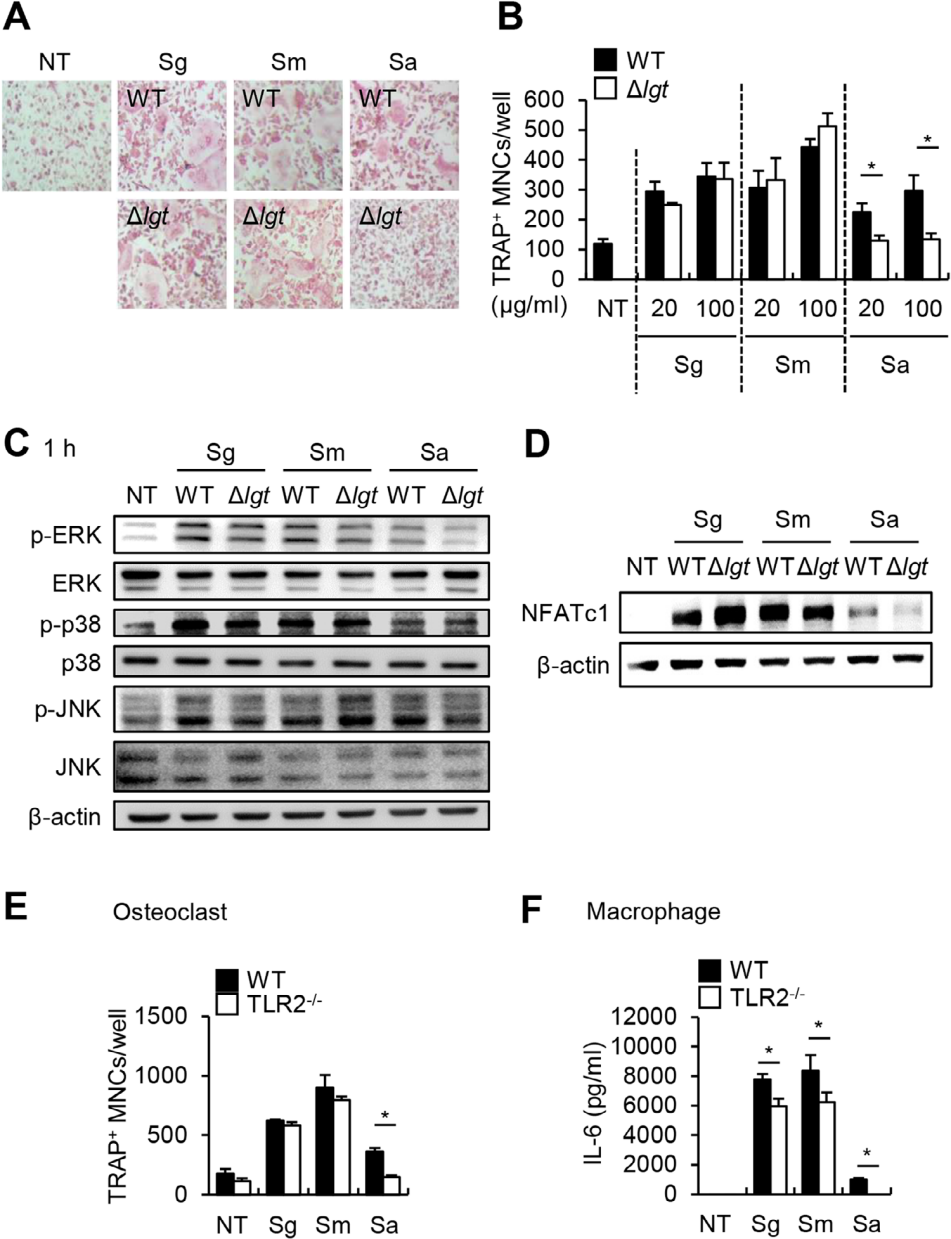
Streptococci augment osteoclast differentiation even in the absence of their lipoproteins. (A, B) Committed osteoclasts were treated with heat‐inactivated *S. gordonii*, *S. mutans*, or *S. aureus* for 24 h. The cells were fixed and subjected to TRAP staining. TRAP‐positive MNCs with three or more nuclei were enumerated through a microscopic analysis. (C) Committed osteoclasts were serum‐starved for 3 h and treated with heat‐inactivated bacteria in the absence of M‐CSF for 1 h. Western blotting was performed using the cell lysates with antibodies against the phosphorylated or non‐phosphorylated forms of MAPKs. Representative immunoblots from two independent experiments are shown. (D) Committed osteoclasts were stimulated with heat‐inactivated bacteria for 24 h. The cell lysates were prepared and subjected to Western blotting with antibodies specific to NFATc1. (E) Committed osteoclasts obtained from WT or TLR2^−/−^ mice were stimulated with heat‐inactivated bacteria for 24 h. The cells were fixed, and TRAP‐positive MNCs were enumerated. (F) BMMs obtained from WT or TLR2^−/−^ mice were stimulated with heat‐inactivated bacteria for 24 h. The cell culture supernatants were analyzed for IL‐6 production using ELISA. One of three similar results is shown. NT, non‐treatment; Sg, heat‐inactivated *S. gordonii*; Sm, heat‐inactivated *S. mutans*; Sa, heat‐inactivated *S. aureus*. Student's *t*‐test was performed to compare each group. *p < 0.05.

### 3.3 Streptococcal Man‐PTSIID Engages With RANK in a Manner Analogous to RANKL

Because both *S. gordonii* and *S. mutans* upregulated osteoclast differentiation in the absence of their lipoproteins, we prepared membrane proteins from WT and ∆*lgt S. gordonii*, *S. mutans*, and *S. aureus* strains to identify the bacterial components. First, we demonstrated that Triton X‐114 extracts from the WT and Δ*lgt* strains of each *S. gordonii* (SgTX) and *S. mutans* (SmTX) induced osteoclastogenesis to similar degrees, whereas Triton X‐114 extracts from the *S. aureus* Δ*lgt* strain (SaTX Δ*lgt*) did not induce osteoclast differentiation, unlike its WT‐derived extract (Figure 3A). Interestingly, the IL‐6 and TNF‐α production levels in the cell culture supernatants were completely diminished in the Δ*lgt* strains of all bacteria, unlike their effects on the osteoclast differentiation (Figure 3B,C), suggesting that the factor driving streptococcus‐induced osteoclastogenesis might not be involved in the production of the pro‐inflammatory cytokines as RANKL. To identify the molecule, we used Q Exactive Plus mass spectrometry to analyze each Triton X‐114 extract. Combining the *in* *silico* analysis of the whole genome databases of *S. gordonii* CH1, *S. mutans* ATCC 25175, and *S. aureus* NCTC 8325, we identified 428 proteins for SgTX WT, 577 proteins for SgTX Δ*lgt*, 657 proteins for SmTX WT, 975 proteins for SmTX Δ*lgt*, 516 proteins for SaTX WT, and 914 proteins for SaTX Δ*lgt* as potential candidate molecules. To identify the bacterial components responsible for the TLR2‐independent osteoclastogenic activity of streptococci, membrane‐associated proteins detected in both *S. gordonii* and *S. mutans* but absent or minimally detected in *S. aureus* were first selected as candidate proteins. These candidates were subsequently compared with the RANK receptor‐binding interface to identify proteins containing RANKL‐like sequences (Table S1). Among those identified proteins, Man‐PTS components were commonly present in *S. gordonii* and *S. mutans* at percentages of 0.77%, 0.89%, 2.53%, 2.65%, 0.01%, and 0.11% for SgTX WT; SgTX Δ*lgt*; SmTX WT; SmTX Δ*lgt*; SaTX WT; and SaTX Δ*lgt*, respectively, in the bacterial Triton X‐114 extracts (Figure 3D). Among the PTS components, those of Man‐PTS accounted for 44.02%, 43.49%, 74.69%, 66.64%, 0%, and 0% for SgTX WT; SgTX Δ*lgt*; SmTX WT; SmTX Δ*lgt*; SaTX WT; SaTX Δ*lgt*, respectively (Figure 3E). Interestingly, ManIID from *S. gordonii* (SgIID) and *S. mutans* (SmIID) showed 42% sequence similarity to mouse RANKL residues 180–193 (Figure 3F). Considering that streptococci have Man‐PTSIID proteins with RANKL‐like sequences and induce osteoclastogenesis in a TLR2‐independent manner, we hypothesized that streptococcal Man‐PTSIID might directly interact with RANK through a RANKL‐like mechanism. Therefore, we performed an SPR assay using peptides containing conserved amino acid regions. SgIID‐N20_5‐24_ and SmIID‐N20_7‐26_ were synthesized based on the sequence analysis in Figure 3F. As shown in Figure 3G, SgIID‐N20_5‐24_ directly bound to RANK with an equilibrium dissociation constant (K_D_) of 9.26 × 10^−7^ M. As for the kinetics, the estimated association (k_on_) and dissociation (k_off_) constants of SgIID‐N20_5‐24_ were 4.79 × 10^3^ M^−1^s^−1^ and 4.44 × 10^−3^ s^−1^, respectively. Similarly, SmIID‐N20_7‐26_ also bound to RANK in a dose‐dependent manner, with a K_D_ of 3.63 × 10^−6^ M, k_on_ of 3.97 × 10^3^ M^−1^s^−1^, and k_off_ of 1.44 × 10^−2^ s^−1^ (Figure 3H), indicating that both SgIID‐N20_5‐24_ and SmIID‐N20_7‐26_ interact directly with the RANK. Taken together, these results suggest that Man‐PTSIID, as a candidate molecule with osteoclastogenic potential in streptococci, signals through RANK engagement.

**FIGURE 3:**
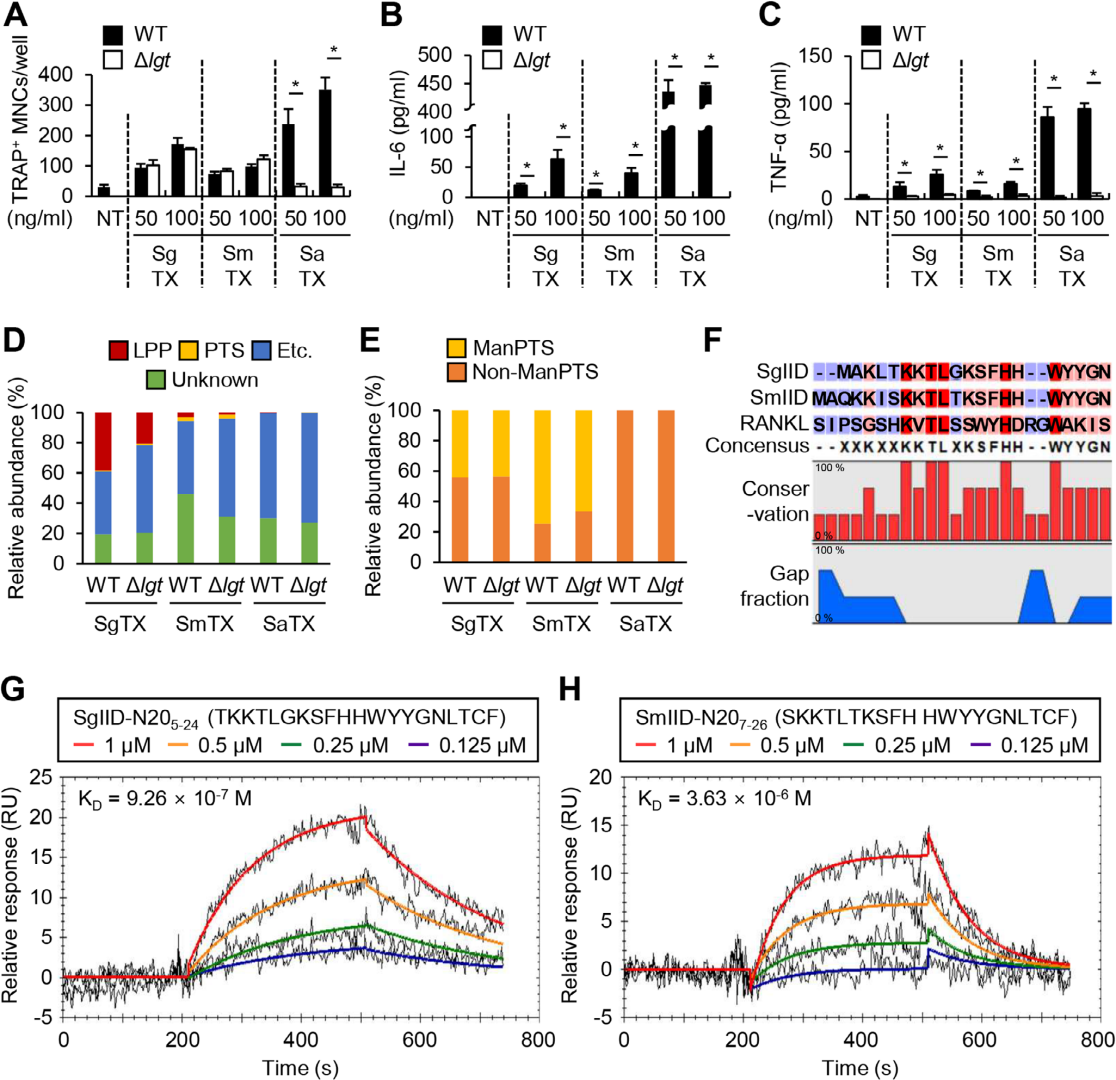
Streptococcal Man‐PTSIID possesses RANKL‐like amino acid sequence and binds to RANK. (A–C) Committed osteoclasts were stimulated with Triton X‐114 extracts of each strain for 24 h. (A) The number of TRAP‐positive MNCs was enumerated through a microscopic analysis. The culture supernatants were analyzed for (B) IL‐6 or (C) TNF‐α production using ELISA. (D, E) Triton X‐114 extracts were analyzed by Q Exactive Plus mass spectrometry. Each Triton X‐114 extract was Coomassie‐stained, and the entire gel lane was analyzed by mass spectrometry. The analyzed proteins were identified by comparing them with the whole genome database of *S. gordonii* CH1, *S. mutans* ATCC 25175, or *S. aureus* NCTC 8325. Relative abundance (%) was calculated as the proportion of identified proteins assigned to each functional category relative to the total number of proteins identified by Q Exactive Plus mass spectrometry. (D) The relative abundances of lipoproteins and PTS proteins among all of the identified proteins are shown. Proteins except lipoproteins and PTS proteins are described as Etc., and proteins that are not identified by Q Exactive Plus mass spectrometry are shown as unknown. (E) The relative abundance of mannose‐PTS proteins among PTS proteins is selectively shown. (F) Man‐PTSIID sequences of *S. gordonii* and *S. mutans* were compared with a structural element overlapping the RANK receptor‐binding interface (180 KVTLSSWYHDRGWA 193). (G, H) After binding RANK to a COOH‐Au chip, (G) SgIID‐N20_5‐24_ or (H) SmIID‐N20_7‐26_ diluted in HBST was run on the chip. The real‐time interaction between RANK and the peptides was monitored using an SPR assay. NT, non‐treatment; SgTX, Triton X‐114 extracts from *S. gordonii*; SmTX, Triton X‐114 extracts from *S. mutans*; SaTX, Triton X‐114 extracts from *S. aureus*; Sg, heat‐inactivated *S. gordonii*; Sm, heat‐inactivated *S. mutans*; Sa, heat‐inactivated *S. aureus*; LPP, Lipoprotein; PTS, Phosphotransferase system; Man‐PTS, Mannose phosphotransferase system. Student's t‐test was performed to compare each group.*P < 0.05.

### 3.4 Man‐PTSIID Directly Binds to RANK, Similar to RANKL

We examined whether Man‐PTSIID interacts with the same surface as RANKL by performing HADDOCK modeling analysis. Full‐length RANK was first docked with Man‐PTSIID from *S. gordonii* and *S. mutans* after the RANKL‐contacting residues of RANK and the conserved N‐terminal region of Man‐PTSIID were defined as active residues. The *S. gordonii* complex reached a HADDOCK score of −85.3 with a buried surface area of 2388 Å² and a Z‐score of −1.6, and the *S. mutans* complex reached a score of −73.2 with a buried surface area of 2189 Å² and a Z‐score of −2.3 (Figure 4A, B). In both models the conserved N‐terminal segment of Man‐PTSIID settled onto the RANK‐binding region that RANKL occupies. Because the full receptor allowed Man‐PTSIID to reach membrane‐proximal regions outside this interface, we refined the model by docking the RANK ectodomain with the N‐terminal region of *S. gordonii* Man‐PTSIID. This run converged on the top‐ranked cluster with a HADDOCK score of −63.2, a buried surface area of 1879 Å², and a Z‐score of −2.6 (Figure 4C). Superposition onto the mouse RANKL‐RANK structure showed that the N‐terminal region of Man‐PTSIID covered the RANKL footprint and engaged RANK residues that RANKL also contacts, including Tyr77, Asp85, Lys86, Lys97, and Trp121. On the Man‐PTSIID side, the conserved residues Trp16, Tyr17, Tyr18, Lys6, and Lys11 inserted into this footprint (Figure 4C). In the mouse RANKL‐RANK complex, the corresponding RANK residues lie within the surface that RANKL reaches through its 180 to 193 segment, which forms a loop in the receptor‐binding interface [24]. The tryptophan and tyrosine of Man‐PTSIID align with the tryptophan and tyrosine that RANKL uses to engage RANK. The overlap indicates that Man‐PTSIID may not bind a separate or allosteric pocket but targets the canonical RANK‐binding region of RANKL. Next, to determine whether PTSIID directly binds to RANK, we examined a pull‐down assay on RANK‐overexpressing HEK293T lysates using full‐length PTSIID protein. As shown in Figure 4D, histidine‐tagged full‐length Man‐PTSIID was detected in RANK immunoprecipitates, suggesting a direct interaction between full‐length Man‐PTSIID and RANK.

**FIGURE 4:**
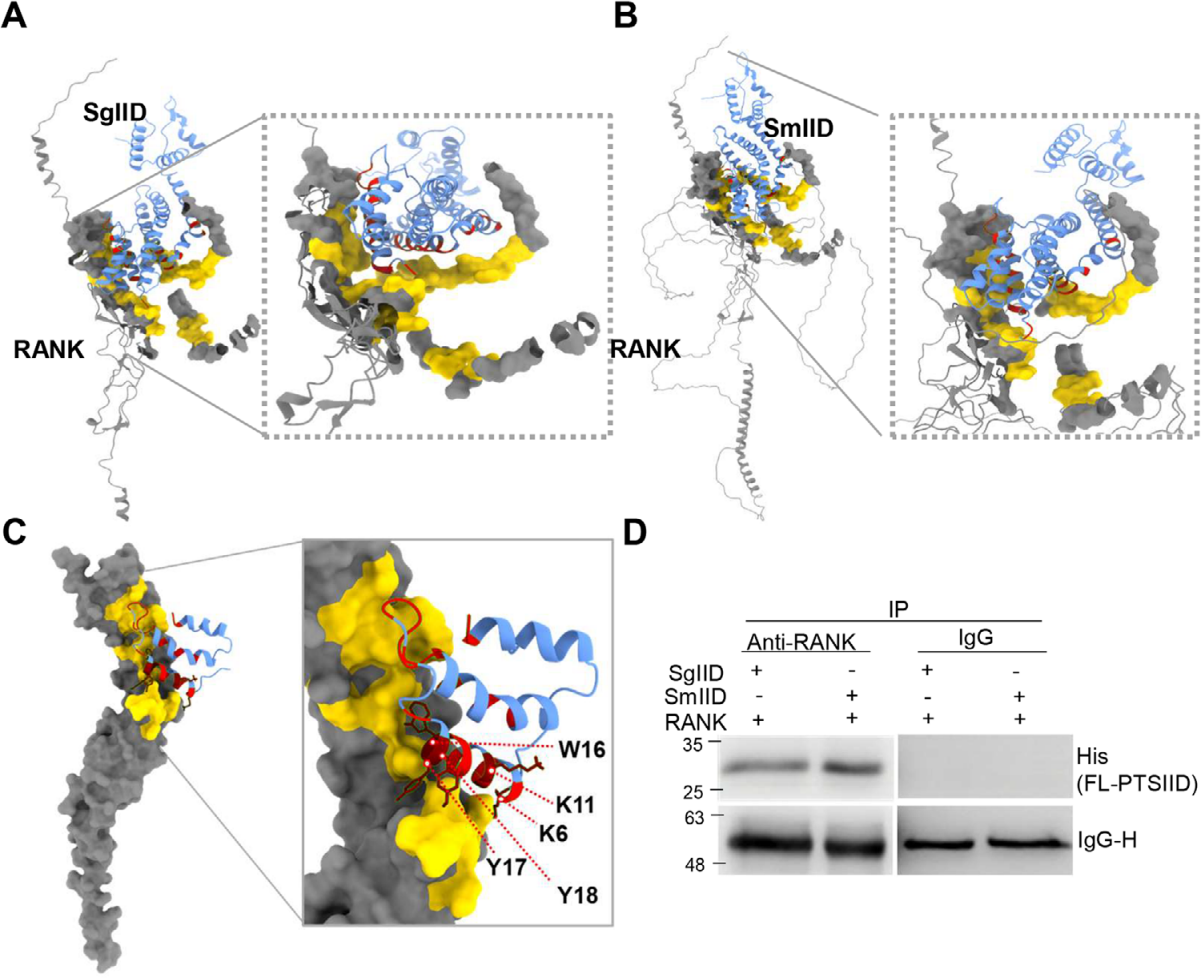
Streptococcal Man‐PTSIID engages the RANK‐binding region of RANKL. (A, B) Full‐length mouse RANK was docked with Man‐PTSIID from (A) *S. gordonii* and (B) *S. mutans* using HADDOCK, with the RANKL‐contacting residues of RANK and the conserved N‐terminal region of Man‐PTSIID were set as active residues. RANK is shown as a gray surface, and Man‐PTSIID as a blue cartoon. The RANK‐binding region of RANKL is colored yellow, and the Man‐PTSIID residues within 5 Å of RANK are colored red. Insets show the interface. (C) The RANK ectodomain was docked with the N‐terminal region of *S. gordonii* Man‐PTSIID and superposed onto the mouse RANKL‐RANK crystal structure (PDB 3ME2). RANK is a gray surface with the RANK‐binding region of RANKL in yellow, and Man‐PTSIID is a blue cartoon. The conserved N‐terminal residues of Man‐PTSIID that insert into the RANKL footprint are shown as red sticks and labeled by single‐letter code (Trp16, Tyr17, Tyr18, Lys6, and Lys11). Residues follow the *S. gordonii* Man‐PTSIID numbering, in which Trp16, Lys6, and Lys11 correspond to Trp18, Lys8, and Lys13 in the *S. mutans* numbering used in the main text. (D) RANK‐expressing HEK293T cells were lysed and incubated with 6×His tagged full‐length Man‐PTSIID proteins. The protein mixture was immunoprecipitated using an anti‐RANK antibody, followed by immunoblot analysis using an anti‐His antibody to detect bound Man‐PTSIID.

### 3.5 Streptococci Induce Osteoclast Differentiation Through a RANK Signaling Pathway

We hypothesized that if RANKL signaling mediates this process, streptococcus‐mediated osteoclastogenesis might be competitively blocked by soluble RANK and OPG, which are a receptor of RANKL and a decoy receptor, respectively. Therefore, we pre‐incubated Triton X‐114 extracts of *S. gordonii*, *S. mutans*, and *S. aureus* with soluble RANK or OPG and then added to committed osteoclasts. Notably, only the osteoclastogenesis induced by the streptococcal Triton X‐114 extracts was reduced following pre‐incubation with soluble RANK or OPG (Figure 5A,B). In addition, similar to RANKL, *S. gordonii* and *S. mutans* increased NF‐κB activity through RANK in a dose‐dependent manner, whereas *S. aureus* did not augment NF‐κB activity (Figure 5C). Next, whole heat‐inactivated *S. gordonii*, *S. mutans*, and *S. aureus* were pre‐incubated with soluble recombinant RANK or OPG before treatment of committed osteoclast precursors. Osteoclast differentiation induced by *S. gordonii* and *S. mutans*, but not *S. aureus*, was significantly reduced in a dose‐dependent manner (Figure 5D–G). Therefore, these neutralization assays suggest that streptococci induce osteoclast differentiation through a RANK signaling pathway, unlike *S. aureus*.

**FIGURE 5:**
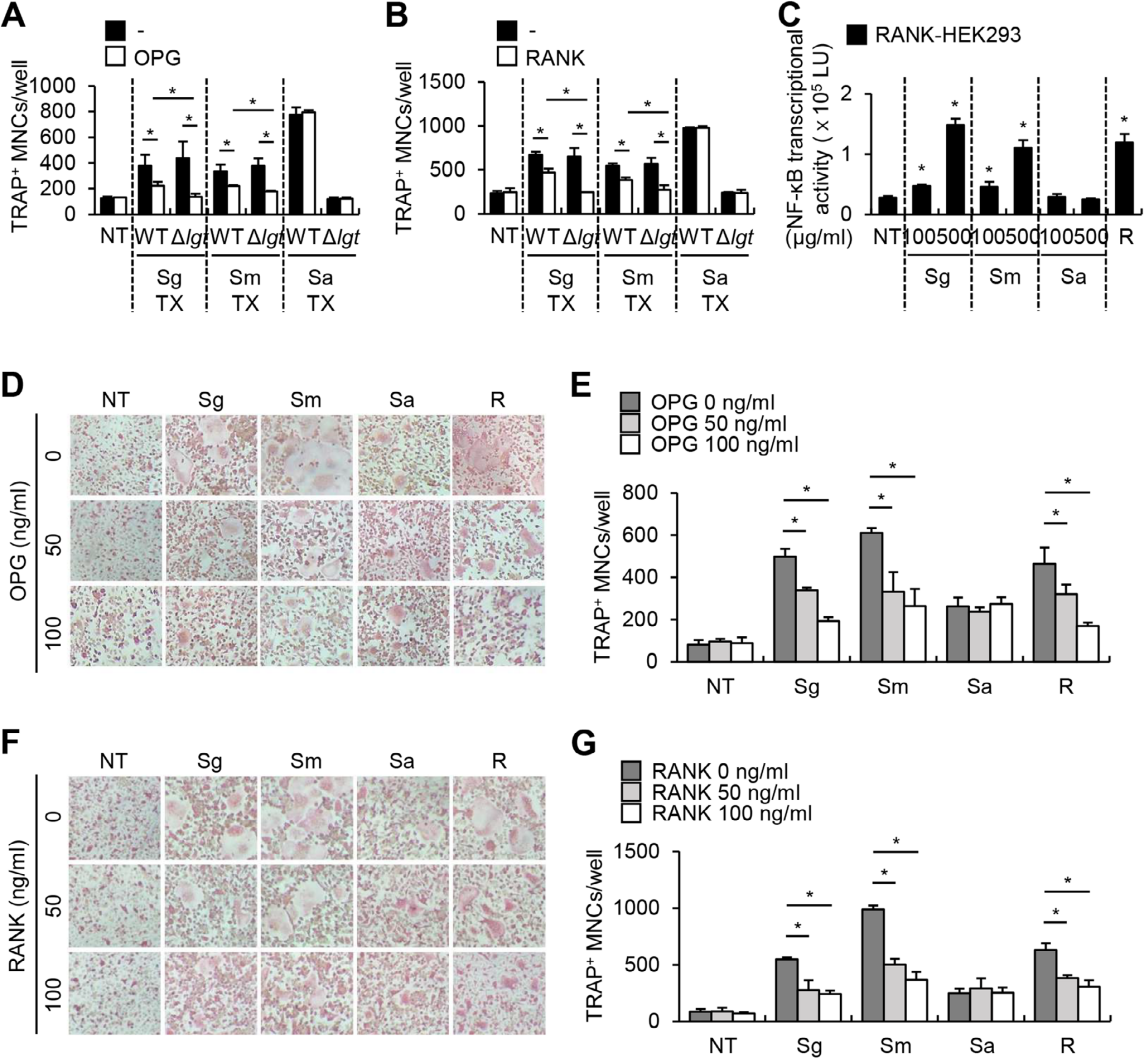
Streptococci induce osteoclast differentiation through a RANK signaling pathway. (A, B) Triton X‐114 extracts were incubated with OPG or soluble RANK for 1 h. The Triton X‐114 extracts incubated with (A) OPG or (B) soluble RANK were administered to committed osteoclasts for 24 h. (C) RANK‐expressing HEK‐293 cells were transfected with luciferase reporter plasmids regulated by the NF‐κB transcription factor for 6 h. The transfected cells were plated on a 96‐well plate and then stimulated with heat‐inactivated bacteria for 24 h. The cells were lysed, and luciferase activity was measured. (D‐G) Heat‐inactivated bacteria or RANKL were incubated with (D,E) OPG or (F,G) soluble RANK for 1 h and then administered to committed osteoclasts. One of three similar results is shown. SgTX, Triton X‐114 extracts from *S. gordonii*; SmTX, Triton X‐114 extracts from *S. mutans*; SaTX, Triton X‐114 extracts from *S. aureus*. Sg, heat‐inactivated *S. gordonii*; Sm, heat‐inactivated *S. mutans*; Sa, heat‐inactivated *S. aureus*; R, RANKL. Student's *t*‐test was performed to compare each group. *p < 0.05.

### 3.6 Man‐PTSIID From Streptococci Directly Promotes Osteoclast Differentiation Through MAPK and NFATc1 Activation

Next, to investigate whether Man‐PTSIID is critical to streptococcus‐induced osteoclast differentiation, we administered the WT and Δ*pts* strains of *S. gordonii* and *S. mutans* to committed osteoclasts. The number of mature osteoclasts in the Δ*pts*‐treated groups was lower than in their WT‐treated counterparts (Figure 6A). Furthermore, compared with the WTs, the Δ*pts* strains of *S. gordonii* and *S. mutans* induced much less phosphorylation of ERK, p38, and JNK (Figure 6B). NFATc1 expression was also diminished in the Δ*pts*‐treated groups, compared with the WTs; this phenomenon was particularly pronounced with *S. mutans* (Figure 6C). To further validate the role of Man‐PTSIID in osteoclast differentiation, we administered recombinant Man‐PTSIID proteins from *S. gordonii* and *S. mutans* to committed osteoclasts. Both Man‐PTSIID proteins potently upregulated the number of TRAP‐positive MNCs in a dose‐dependent manner (Figure 6D, Figure S1A). Man‐PTSIID proteins also increased the phosphorylated forms of MAPKs (ERK, p38, and JNK) in a time‐dependent manner (Figure 6E), and NFATc1 was potently upregulated by Man‐PTSIID proteins (Figure 6F). To determine whether Man‐PTSIID alone could induce de novo osteoclast differentiation, naïve BMMs were treated with recombinant Man‐PTSIID in the absence or presence of RANKL. Man‐PTSIID alone did not trigger osteoclast differentiation. Moreover, co‐treatment with Man‐PTSIID and RANKL failed to augment RANKL‐induced osteoclastogenesis and instead resulted in reduced osteoclast differentiation compared with RANKL alone (Figure 6G, Figure S1B). In contrast, in RANKL‐primed committed osteoclast precursors, co‐treatment with RANKL and Man‐PTSIID further enhanced osteoclast differentiation (Figure 6H), with a particularly marked increase in giant multinucleated osteoclasts containing ≥20 nuclei (Figure 6I, Figure S1C). In addition, pretreatment with soluble OPG or RANK attenuated Man‐PTSIID‐induced osteoclast differentiation, indicating the functional involvement of the RANK signaling pathway. (Figure 6J). Next, to examine the direct in vivo effects of Man‐PTSIID proteins, collagen sheets soaked with recombinant Man‐PTSIID proteins were implanted on mouse calvaria. Recombinant Man‐PTSIID proteins from both *S. gordonii* and *S. mutans* potently augmented bone resorption and the TRAP‐positive areas of mouse calvaria (Figure 6K,L). Moreover, both SgIID‐N20_5‐24_ and SmIID‐N20_7‐26_ potently augmented the number of TRAP‐positive MNCs (Figure 6M), which was diminished by OPG or RANK treatment (Figure 6N). To further identify the minimal functional region required for osteoclastogenic activity, we synthesized peptides with fewer amino acids: SmIID‐N11_8‐18_ (KKTLTKSFHHW), SmIID‐N7_8‐14_ (KKTLTKS), and SmIID‐N5_10‐14_ (TLTKS). SmIID‐N11_8‐18_ increased osteoclastogenesis as much as SmIID‐N20_7‐26_, but SmIID‐N7_8‐14_ induced much less osteoclast differentiation, and SmIID‐N5_10‐14_ did not augment osteoclastogenesis at all (Figure 6O). Collectively, Man‐PTSIID from streptococci is a potent osteoclast differentiation and bone resorption factor that activates MAPK and NFATc1 signaling cascades through RANK engagement.

**FIGURE 6:**
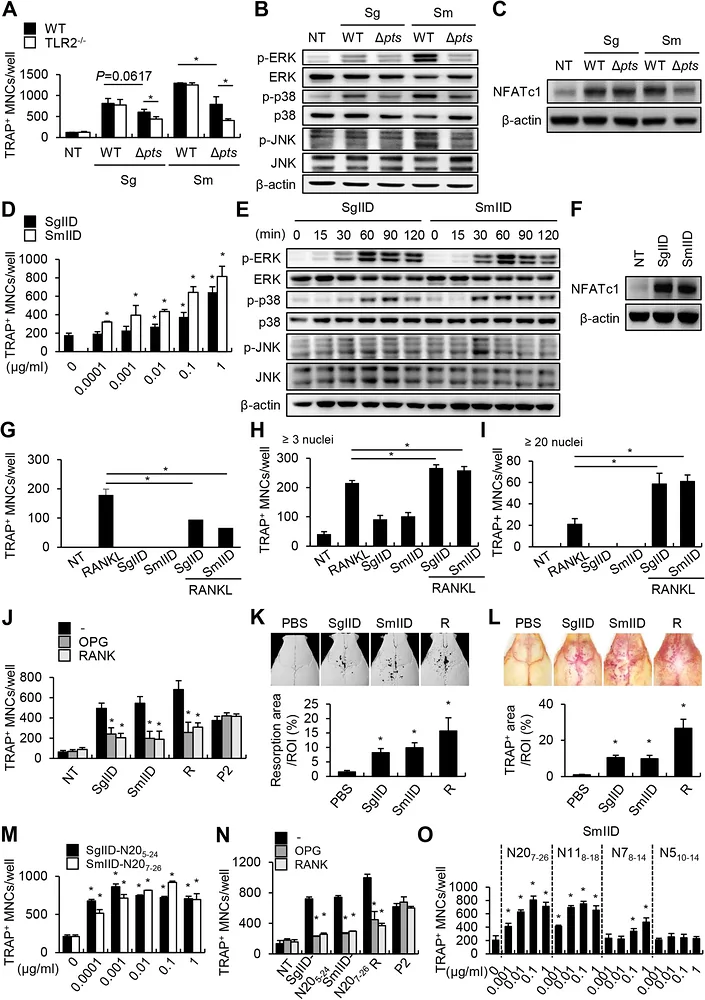
Streptococcal Man‐PTSIID increases osteoclastogenesis through MAPK and NFATc1 activation. (A) Committed osteoclasts from WT or TLR2^−/−^ mice were stimulated with heat‐inactivated WT and Δ*pts S. gordonii* and *S. mutans*. After 24 h, the mature osteoclasts were fixed, and TRAP‐positive MNCs were enumerated by microscopic analysis. (B) After 3 h of serum‐starvation, committed osteoclasts were treated with heat‐inactivated bacteria for 1 h. MAPK phosphorylation was determined by Western blotting using antibodies specific to MAPK. (C) Heat‐inactivated bacteria were administered to committed osteoclasts for 24 h. NFATc1 expression was determined by Western blotting. (D) Committed osteoclasts were stimulated with Man‐PTSIID in 10‐fold serial dilutions for 24 h. (E) Committed osteoclasts were serum‐starved for 3 h and then stimulated with Man‐PTSIID for 15, 30, 60, 90, or 120 min. The cells were lysed and subjected to Western blotting using antibodies specific against MAPK. (F) Committed osteoclasts were treated with Man‐PTSIID for 24 h. Western blotting was performed using antibodies specific to NFATc1. (G) BMMs were treated with Man‐PTSIID in the presence or absence of RANKL for 3 days. (H, I) Committed osteoclasts were stimulated with Man‐PTSIID in the presence or absence of RANKL for 16 h. (J) SgIID, SmIID, RANKL, or Pam2CSK4 were pre‐incubated with soluble recombinant RANK or OPG (100 ng/mL) for 1 h, and the mixtures were directly applied to committed osteoclast precursors for 24 h. TRAP‐positive MNCs were enumerated. (K) Collagen sheets soaked with 50 µg of Man‐PTSIID were implanted on mouse calvaria (*n* = 3 per group). After 7 days, the calvarial bone was scanned by micro‐CT. The resorption area was analyzed using ImageJ. (L) The calvaria were stained for TRAP, and the TRAP‐positive area was analyzed using ImageJ. (M) Committed osteoclasts were stimulated with SgIID‐N20_5‐24_ or SmIID‐N20_7‐26_ for 24 h, and TRAP‐positive MNCs were enumerated. (N) SgIID‐N20_5‐24_, SmIID‐N20_7‐26_, RANKL, or Pam2CSK4 was incubated with OPG or RANK for 1 h and administered to committed osteoclasts for 24 h. TRAP‐positive MNCs with three or more nuclei were enumerated. (O) Committed osteoclasts were stimulated with SmIID‐N20_7‐26_, SmIID‐N11_8‐18_, SmIID‐N7_8‐14_, or SmIID‐N5_10‐14_. After 24 h, the cells were fixed and TRAP‐stained. Sg, heat‐inactivated *S. gordonii*; Sm, heat‐inactivated *S. mutans*; SgIID, recombinant protein of *S. gordonii* CH1 Man‐PTSIID; SmIID, recombinant protein of *S. mutans* ATCC 25175 Man‐PTSIID; P2, Pam2CSK4; ROI, region of interest. Student's *t*‐test was performed to compare each group. *p < 0.05.

### 3.7 Streptococcal Species Commonly Induce Osteoclastogenesis Through a RANK Signaling Pathway

To determine whether other streptococcal species also possess PTSIID with RANKL amino acid sequences (KVTLSSWYHDRGWA), an amino acid sequence similarity analysis was performed using CLC Sequence Viewer. When we compared the amino acid sequences of streptococcal Man‐PTSIID, all of the tested oral streptococci (*S. gordonii*, *S. mutans*, *S. oralis*, *S. sobrinus*, *S. sanguinis*, and *S. mitis*) had conserved amino acid sequences in the N‐terminus (Figure 7A). Those oral streptococci induced osteoclastogenesis in a TLR2‐independent manner, while other Gram‐positive bacteria (*S. aureus* USA300, *S. epidermidis*, *B. subtilis*, and *L. plantarum*) augmented osteoclast differentiation in a TLR2‐dependent manner (Figure 7B). Interestingly, OPG and RANK inhibited the osteoclastogenesis induced by streptococci, but not that induced by other Gram‐positive bacteria (Figure 7C). Collectively, these results suggest that conserved N‐terminal Man‐PTSIID sequences may contribute to the RANKL‐like osteoclastogenic activity shared by multiple oral streptococcal species.

**FIGURE 7:**
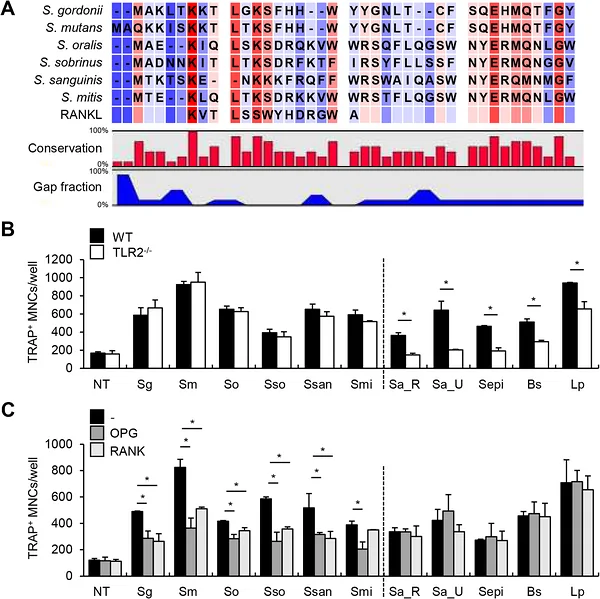
Streptococcal species induce osteoclastogenesis through a RANK signaling pathway. (A) ManIID sequences of *S. gordonii*, *S. mutans*, *S. oralis*, *S. sobrinus*, *S. sanguinis*, and *S. mitis* were compared with a structural element overlapping the RANKL receptor‐binding interface (180 KVTLSSWYHDRGWA 193). (B) Committed osteoclasts from WT or TLR2^−/−^ mice were stimulated with heat‐inactivated *S. gordonii*, *S. mutans*, *S. oralis*, *S. sobrinus*, *S. sanguinis*, *S. mitis*, *S. aureus* RN4220, *S. aureus* USA300, *S. epidermidis*, *B. subtilis*, or *L. plantarum* for 24 h. The mature osteoclasts were TRAP‐stained and enumerated by microscopy. (C) Heat‐inactivated bacteria were incubated with OPG or soluble RANK for 1 h and then administered to committed osteoclasts for 24 h. The mature osteoclasts were TRAP‐stained and enumerated by microscopy. One of three similar results is shown. NT, non‐treatment; Sg, heat‐inactivated *S. gordonii*; Sm, heat‐inactivated *S. mutans*; So, heat‐inactivated *S. oralis*; Sso, heat‐inactivated *S. sobrinus*; Ssan, heat‐inactivated *S. sanguinis*; Smi, heat‐inactivated *S. mitis*; Sa_R, heat‐inactivated *S. aureus* RN4220; Sa_U, heat‐inactivated *S. aureus* USA300; Sepi, heat‐inactivated *S. epidermidis*; Bs, heat‐inactivated *B. subtilis*; Lp, heat‐inactivated *L. plantarum*; *L. ruminis*, heat‐inactivated *L. ruminis*. Student's *t*‐test was performed to compare each group. *p < 0.05.

## 4 Discussion

In this study, we newly identified a novel streptococcal bone‐resorbing protein, Man‐PTSIID, which operates through a mechanism distinct from previously known pathogenic pathways. Although the lipoproteins of Gram‐positive bacteria have been understood to trigger potent bone destruction via the TLR2 signaling pathway [15], streptococci induce bone resorption through a RANK‐dependent pathway, deviating from the TLR2 mechanism. Streptococcal Man‐PTSIID exhibit sequence similarity with RANKL, allowing them to interact with RANK and enhance osteoclast differentiation, ultimately contributing to bone destruction. In addition, streptococci generate hydrogen peroxide that can induce apoptotic cell death [25]. The combined effects of direct cytotoxicity, inflammatory signaling, and RANK activation by the RANK‐related osteoclastogenic factor, Man‐PTSIID, contribute to bone destruction in conditions such as osteomyelitis, periodontal disease, and prosthetic joint infections. Further investigation into this newly identified mechanism may provide important insights for the development of strategies to control streptococcal bone disorders and regulate excessive osteoclastogenesis induced by streptococcal infection.

Streptococcal lipoproteins were not responsible for bone destruction, unlike the lipoproteins of other Gram‐positive bacteria. In general, lipoproteins are the major bone‐destructive factor of Gram‐positive bacteria [15, 16]. For example, *S. aureus* does not augment bone resorption without their lipoproteins. They increase osteoclastogenesis through a TLR2‐MyD88 signaling pathway [15]. Unlike other Gram‐positive bacteria, the Δ*lgt* strains of *S. gordonii* and *S. mutans* induced bone destruction and osteoclast differentiation to the same extent as their WT counterparts in both in vivo and in vitro experiments. Furthermore, in both WT and TLR2^−/−^ committed osteoclasts, *S. gordonii* and *S. mutans* upregulated osteoclastogenesis. These findings suggest that the primary pathway of streptococcal osteoclast differentiation is not associated with lipoproteins.

The current study showed that the major bone‐resorbing factor in streptococci is a RANK‐binding osteoclastogenic factor, Man‐PTSIID. The Δ*pts* strains of *S. gordonii* and *S. mutans* induced far less osteoclast differentiation than their WT counterparts. In addition, recombinant Man‐PTSIID proteins upregulated osteoclast differentiation in in vitro experiments. It seems that Man‐PTSIID engages with RANK to upregulate osteoclastogenesis. The affinity between RANK and RANKL plays a crucial role in endogenous osteoclast differentiation and the onset of bone diseases [26]. Likewise, in our data, peptides composed of the conserved sequence, SgIID‐N20_5‐24_ and SmIID‐N20_7‐26_, physically bound RANK and augmented osteoclast differentiation. Considering the correlation between RANK–RANKL binding affinity and the degree of osteoclastogenesis [26], it is reasonable that RANKL‐like Man‐PTSIID bound RANK and triggered osteoclast differentiation. In addition, although native RANKL activates RANK through homotrimer formation [26], whether Man‐PTSIID similarly requires oligomerization remains unknown. While our structural prediction and binding analyses support a direct interaction between Man‐PTSIID and RANK, further studies employing RANK/NF‐κB reporter assays, competition‐binding assays, and binding motif‐directed mutagenesis will be necessary to fully elucidate the molecular mechanism of RANK activation by Man‐PTSIID. Our findings also suggest that the biological activity of Man‐PTSIID is dependent on the differentiation stage of osteoclasts, although the mechanism underlying this stage‐dependent effect remains unclear. Furthermore, studies using RANKL‐deficient models will be important to determine whether streptococci‐induced bone loss is dependent on RANKL signaling in vivo. Collectively, these results suggest that Man‐PTSIID functions as a novel, streptococcal RANK‐binding osteoclastogenic factor that contributes to osteoclast differentiation following RANKL priming and plays an important role in bone pathology during streptococcal infections.

Bacteria with Man‐PTSIID that have amino acid sequences similar to those in streptococci enhanced bone resorption via a RANK‐dependent pathway. The amino acid sequences of Man‐PTSIID in different streptococcal species show considerable similarity. In particular, these bacteria often have a conserved sequence, KXTLXKSXXXW, that resembles the receptor binding site of RANKL at their N‐terminus. *L. ruminis*, which possesses Man‐PTSIID with an amino acid sequence similar to that of streptococci, also augmented osteoclast differentiation through a RANK signaling pathway. On the contrary, other Gram‐positive bacteria without Man‐PTSIID induce osteoclastogenesis in a TLR2‐dependent manner. Therefore, RANK‐activating osteoclast differentiation appears to be unique to streptococci and other bacteria with Man‐PTSIID amino acid sequences similar to those of streptococci.

The six highly conserved amino acids in streptococci, Man‐PTSIID^K8, T10, L11, K13, S14, W18^, seem to play an essential role in RANK binding and osteoclastogenesis. In our study, the deletion of Man‐PTSIID^W18, K8^ reduced the degree of osteoclastogenesis, indicating that those amino acids are crucial for streptococcal bone destruction. Further deletion studies, particularly focusing on the critical six amino acids, could provide valuable insights into streptococcus‐induced bone resorption. Based on that understanding, we look forward to developing peptide‐based therapeutics against streptococci. Peptide drugs are particularly advantageous due to their specific biochemical properties and weak toxicity [27, 28]. These findings may serve as a foundation for the design of specific anti‐streptococcal drugs.

In summary, this study demonstrates that RANK‐binding Man‐PTSIID proteins, rather than lipoproteins, are the primary pathogenic molecules that promote osteoclast differentiation and mediate streptococcal bone destruction. This previously unrecognized mechanism highlights a novel way by which streptococci contribute to bone resorption. Our findings further suggest that oral streptococci may act as virulence factors promoting osteoporotic bone loss in periodontal and other oral diseases, thereby expanding the current understanding of their pathogenic potential. These insights underscore the importance of targeting streptococcal Man‐PTSIID as a potential therapeutic strategy for bone disorders associated with streptococcal infections.

## Author Contributions

**Chaeyeon Park**: Writing – original draft, Writing – review & editing, Visualization, Validation, Methodology, Formal analysis, Data curation. **Yeongkag Kwon**, Yeonjin Lim, Jeong Woo Park, **Hyun Jung Ji**, **Dongwook Lee**: Writing – reviewing, Methodology, Investigation, Formal analysis. **Sung‐Ho Yun**, **Sungho Jeong**, **Ho Seong Seo**, **Kun Cho**: Writing – reviewing, Methodology, Investigation. **Cheol‐Heui Yun**: Writing – review & editing, Data curation. **Ok‐Jin Park** ^:^ Writing – original draft, Writing – review & editing, Supervision, Conceptualization, Data curation, Formal analysis, Validation. **Seung Hyun Han**: Writing – original draft, Writing – review & editing, Supervision, Funding acquisition, Conceptualization, Data curation.

## Conflicts of Interest

The authors declare no conflicts of interest.

## Supporting information




**Supporting File 1**: advs77282‐sup‐0001‐SuppMat.xlsx.

**Supporting File 2**: advs77282‐sup‐0002‐SuppMat.docx.

**Supporting File 3**: advs77282‐sup‐0003‐SuppMat.docx.

## Data Availability

The raw mass spectrometry data generated in this study have been deposited in the Zenodo repository under accession number https://doi.org/10.5281/zenodo.17247173. The data that support the findings of this study are available from the corresponding author upon reasonable request.
