# Supplementary material for: Streptococcal Mannose Phosphotransferase System Component IID Is a Novel RANK‐Binding Osteoclastogenic Factor
Source: Adv Sci (Weinh). 2026 Aug 19;13(62):e77282. doi: 10.1002/advs.77282 (PMC13488869; doi:10.1002/advs.77282)
Supplement: Supplementary file 2 [file ADVS-13-e77282-s003.docx]

**TABLE 1. Proteins with RANKL-like sequences**

| Bacteria | Protein | MOI (%) | Identity (%) |
| --- | --- | --- | --- |
| *S. gordonii* | ABC transporter, substrate-binding protein SP0092 | 0.4722 | 14 |
|  | ABC transporter, permease protein | 0.0055 | 7 |
|  | sortase B | 1.3969 | 35 |
|  | 50S ribosomal protein L18 | 0.0405 | 14 |
|  | 50S ribosomal protein L5 | 0.0239 | 28 |
|  | PTS system, mannose/fructose/sorbose family, IID component | 0.0787 | 7 |
|  | PTS system, IIBC component | 0.0239 | 14 |
|  | PTS system, fructose(mannose)-specific IID | 0.0069 | 42 |
| *S. mutans* | foldase protein PrsA | 0.4108 | 14 |
|  | ABC transporter | 1.2965 | 7 |
|  | oligopeptide ABC transporter substrate-binding protein | 0.3138 | 35 |
|  | putative ABC transporter, phosphate-binding protein | 0.3001 | 50 |
|  | ABC transporter ATP-binding protein | 0.0936 | 14 |
|  | glutamine ABC transporter ATP-binding protein | 0.0479 | 35 |
|  | amino acid ABC transporter ATP-binding protein | 0.0474 | 78 |
|  | putative ABC transporter, ATP-binding protein, MsmK-like protein | 0.0345 | 28 |
|  | ABC transporter membrane protein | 0.0316 | 21 |
|  | ABC transporter ATP-binding protein | 0.0067 | 7 |
|  | putative ABC transporter integral membrane protein | 0.0058 | 50 |
|  | ABC transporter permease | 0.0058 | 7 |
|  | polysaccharide ABC transporter ATP-binding protein | 0.0042 | 14 |
|  | ABC transporter membrane protein subunit and ATP-binding protein | 0.0021 | 64 |
|  | 50S ribosomal protein L5 | 0.2297 | 28 |
|  | PTS system mannose family transporter subunit IID | 0.2206 | 7 |
|  | putative PTS system mannose-specific IID component | 0.0404 | 42 |
|  | PTS system glucose/maltose-specific transporter subunit IIBCA | 0.0362 | 14 |
|  | PTS system trehalose-specific transporter subunits IIBCA | 0.0208 | 14 |
|  | PTS system, mannitol-specific enzyme IIBC component | 0.0029 | 28 |
|  | preprotein translocase subunit SecA | 0.0208 | 14 |
