# Supplementary material for: Streptococcal Mannose Phosphotransferase System Component IID Is a Novel RANK‐Binding Osteoclastogenic Factor
Source: Adv Sci (Weinh). 2026 Aug 19:e77282. Online ahead of print. doi: 10.1002/advs.77282 (PMC13488869; doi:10.1002/advs.77282)
Supplement: Supplementary file 1 [file ADVS-9999-e77282-s002.docx]

**Streptococcal mannose phosphotransferase system component IID is a novel RANK-binding osteoclastogenic factor**

**Supplementary Figure 1. Man-PTSIID preferentially promotes osteoclast maturation after lineage commitment.** (A) Committed osteoclast precursors were treated with *S. gordonii* or *S. mutants* PTSIID for 24 h with M-CSF. (B) BMMs were treated with SgIID or SmIID in the presence or absence of RANKL for 3 days. (C) Committed osteoclast precursors were treated with SgIID or SmIID in the presence or absence of RANKL for 16 h. TRAP-positive MNCs with three or more nuclei were enumerated through a microscopic analysis. NT, non-treatment; Sg, *S. gordonii*; Sm, *S. mutans*; *S. gordonii* Man-PTSIID; SmIID, *S. mutans* Man-PTSIID.
